# Joint system relaxometry (JSR) and Crámer‐Rao lower bound optimization of sequence parameters: A framework for enhanced precision of DESPOT T_1_ and T_2_ estimation

**DOI:** 10.1002/mrm.26670

**Published:** 2017-03-16

**Authors:** Rui Pedro A.G. Teixeira, Shaihan J. Malik, Joseph V. Hajnal

**Affiliations:** ^1^ Division of Imaging Sciences and Biomedical Engineering King's College London London United Kingdom; ^2^ Centre for the Developing Brain King's College London London United Kingdom

**Keywords:** relaxometry, CRLB, DESPOT

## Abstract

**Purpose:**

This study aims to increase the precision of single‐compartment DESPOT relaxometry by two means: (i) a joint system relaxometry (JSR) approach that estimates parameters in a single step using all available data; and (ii) optimizing acquisition parameters by deploying a robust design tool based on the Crámer‐Rao lower bound (CRLB).

**Methods:**

Following the development of the analysis and design capabilities, phantom and four in vivo subject experiments were performed to compare directly the precision achieved with DESPOT and JSR estimation using published protocols and protocols designed using a proposed CRLB framework.

**Results:**

Experimental data demonstrate JSR's ability to decrease relaxometry estimation variance. Phantom results show 72 to 77% improvement using the same data as conventional DESPOT. This is further improved to 81 to 87% using optimal parameters. Both experiments show systematic bias depending on the acquisition parameters used, which are shown to be highly reproducible and to vary with different magnetization transfer conditions.

**Conclusions:**

Compared with DESPOT, JSR produces reproducible relaxation maps with improved precision. Further improvement was achieved using CRLB as a protocol design tool. With this combined approach, it is possible to achieve submillimeter maps of $\rho ,T_{1},T_{2},\;\text{and}\;B_{0}$ in an 11‐min examination, making the approach appealing for potential clinical use. **Magn Reson Med 79:234–245, 2018. © 2017 The Authors Magnetic Resonance in Medicine published by Wiley Periodicals, Inc. on behalf of International Society for Magnetic Resonance in Medicine. This is an open access article under the terms of the Creative Commons Attribution License, which permits use, distribution and reproduction in any medium, provided the original work is properly cited.**

## INTRODUCTION

Despite the continued dominance of qualitative assessment in clinical applications of MRI, the ability to probe tissue relaxation times $T_{1}$ and $T_{2}$ has proven to be useful—particularly in detecting and understanding neurological diseases 1, 2. Unfortunately, “gold standard” methods for estimating $T_{1}$ and $T_{2}$ are 2D acquisitions based on spin‐echo (SE) methods, which require long acquisition times and have been shown to produce variable results across different studies 3, hindering their application in clinical settings 4. Gradient‐echo methods such as the variable flip angle (VFA) method 5, or driven equilibrium single pulse observation of $T_{1}$ and $T_{2}$ (DESPOT1/2) 6 were previously shown to generate 1‐mm^3^ 3D maps of the human brain in clinically feasible times 6, 7. However, they have been reported to produce a systematic offset when compared with gold standard methods and, to the authors' knowledge, a common ground between both has yet to be found 3, 8.

The DESPOT1/2 approach makes use of spoiled gradient echo (SPGR) and balanced steady‐state free precession (bSSFP) sequences over a range of varying flip angles (FAs) at fixed repetition times (TRs), to estimate $T_{1}$ and $T_{2}$ of each measured voxel. The standard fitting procedure 6 obtains $T_{1}$ from a linearized Ernst relationship using SPGR data alone, and then feeds the $T_{1}$ value into a linearized bSSFP model to estimate $T_{2}\;$ A DESPOT2 approach that avoids linearization of the equations (DESPOT‐FM) was also proposed, to estimate the underlying field map 9. Recently, multicomponent signal models have also been proposed 10, 11, and an interesting discussion regarding their estimation stability based on both Monte Carlo 12 and Cramér‐Rao lower bound (CRLB) methods 13 can be found elsewhere 12, 13, 14 (the CRLB is a statistical tool that predicts the minimum obtainable variance of the estimated model parameters given the noise properties of the measurement experiment 13, 15, 16). However, a limited amount of work has been presented 17, 18, 19, 20 regarding the evaluation of the single‐compartment model for the range of relaxation times found in the human brain. This study is concerned with optimizing the estimation precision, and thereby the efficiency of single‐compartment DESPOT‐style methods. It is divided into two main topics. First, a joint system relaxometry (JSR) approach is adopted, in which full (ie, not linearized) models for both SPGR and bSSFP signals are simultaneously evaluated, maximizing the information that is used to estimate the relaxation parameters. Second, following the work presented by Lankford and Does 13, a way to optimize the JSR based on the CRLB framework is proposed. This allows the selection of optimized acquisition protocols for tissues of interest. The resulting framework has been tested on a phantom and in vivo to determine the performance benefits that can be achieved.

## THEORY

### DESPOT Theory

Originally called variable nutation angle 21, the DESPOT nomenclature was first introduced by Homer and Beevers in 1985 21 and expanded to DESPOT1/2 by Deoni et al in 2003, when the use of the bSSFP sequence to map $T_{2}$ information after $T_{1}$ had been assessed was suggested 6. However, this ignores both $T_{2}$ or $T_{2}^{*}$ dependency of the SPGR signals as well as $T_{1}$ information present in the bSSFP curves. Both effects can be taken into consideration by carefully stating the signal evolution for different acquisition parameters. The expected SPGR signal equation, *S_SPGR_*, adopted in this work, is given by 6, 22, 23 as follows:

(1) $$S_{SPGR}\left(\alpha ,T_{R}^{\text{SPGR}}\right)=\frac{1-E_{1}(T_{R}^{\text{SPGR}})}{1-E_{1}(T_{R}^{\text{SPGR}})\cos\left(\kappa \alpha \right)}\sin\left(\kappa \alpha \right)e^{-\frac{T_{E}^{SPGR}}{T_{2}}}e^{-\frac{T_{E}^{SPGR}}{T_{2}^{\prime }}}e^{i2\pi \Delta \Omega T_{E}^{SPGR}}$$

whereas the bSSFP signal, *S_bSSFP_*, is described by 22, 24, 25 as follows:

(2) $$S_{SSFP}\left(\alpha ,T_{R}^{\text{bSSFP}}\right)=\left[M_{x}^{SS}\left(\alpha ,T_{R}^{\text{bSSFP}}\right)+iM_{y}^{SS}\left(\alpha ,T_{R}^{\text{bSSFP}}\right)\right]e^{-\frac{T_{E}^{bSSFP}}{T_{2}}}e^{i2\pi \Delta \Omega T_{E}^{bSSFP}}$$

where

(3) $$M_{x}^{SS}(\alpha ,T_{R}^{\text{bSSFP}})=\left(1-E_{1}\left(T_{R}^{\text{bSSFP}}\right)\right)\frac{E_{2}\left(T_{R}^{\text{bSSFP}}\right)\text{sin}\left(\kappa \alpha \right)\sin\left(\beta \right)}{d}$$

(4) $$M_{y}^{SS}(\alpha ,T_{R}^{\text{bSSFP}})=\left(1-E_{1}\left(T_{R}^{\text{bSSFP}}\right)\right)\frac{\left(1-E_{2}\left(T_{R}^{\text{bSSFP}}\right)\cos\left(\beta \right)\right)\text{sin}(\kappa \alpha )}{d}$$

and

(5) $$d=\left(1-E_{1}\left(T_{R}^{\text{bSSFP}}\right)\cos\left(\kappa \alpha \right)\right)\left(1-E_{2}\left(T_{R}^{\text{bSSFP}}\right)\cos\left(\beta \right)\right)-E_{2}\left(T_{R}^{\text{bSSFP}}\right)(E_{1}\left(T_{R}^{\text{bSSFP}}\right)-\cos\left(\kappa \alpha \right))(E_{2}\left(T_{R}^{\text{bSSFP}}\right)-\cos\left(\beta \right))$$

Equations [1] to [5] describe a full magnetization model, which we refer to as joint system relaxometry, to distinguish it from the commonly used sequential procedure. Here, $T_{R}^{SPGR}$, $T_{E}^{SPGR}$ and $T_{R}^{bSSFP}$, and $T_{E}^{bSSFP}$ are the repetition and echo times of the SPGR and bSSFP sequences; $T_{2}^{\prime }$ is defined by 1/ $T_{2}^{*}$ = 1/T_2_ + 1/ $T_{2}^{\prime }$; α is the FA modulated by a (dimensionless) transmit field inhomogeneity *κ*; $E_{1}(T_{R}^{\text{SPGR},\text{bSSFP}})=\text{exp}(-T_{R}^{\text{SPGR},\text{bSSFP}}/T_{1})$; and $\beta =2{\pi \Delta \Omega T}_{R}^{\text{bSSFP}}+\phi_{\text{RF}}$ is the off‐resonance dephasing angle in radians, resulting from frequency offset ΔΩz(Hz) plus the rotation induced by incrementing the phase, $\phi_{\text{RF}},$ of the radiofrequency (RF) pulse at each $T_{R}^{\text{bSSFP}}$ 22. Finite RF pulse duration ( $T_{\text{RF}}$) was considered by following the correction proposed in 8, 25 and setting $E_{2}=\text{exp}(-(T_{R}-\zeta_{\beta }\frac{T_{\text{RF}}}{T_{R}^{\text{bSSFP}}})/T_{2})$ with $\zeta_{\beta }=\zeta \cos^{2}\left[\frac{\beta }{2}\left(1-(1-\zeta )\frac{T_{\text{RF}}}{T_{R}^{\text{bSSFP}}}\right)\right]$ and $\zeta \approx 0.68-0.125\left(1+\frac{T_{\text{RF}}}{T_{R}^{\text{bSSFP}}}\right)\frac{T_{2}}{T_{1}}\;$ Note that the two sequence types need not have the same repetition and echo times as each other. $M_{x}^{SS}$ and $M_{y}^{SS}$ correspond, respectively, to bSSFP real and imaginary steady‐state components. In Equations [1] to [5] we adopted the convention that all signals are normalized by the proton density, $M_{0}$ as all measured signals have a scanner‐dependent scaling factor that must be explicitly accommodated during fitting (see “Methods” section). Both SPGR (Eq. [1]) and bSSFP (Eqs. [(2), (3), (4), (5)]) signal equations can be immediately derived from the Bloch equations as described elsewhere 22, 25.

The proposed JSR approach requires careful balance between SPGR and bSSFP signal intensities during readout periods. Although possible to compute the echo‐time decay from the magnetization before the RF pulse, using the magnetization immediately after the excitation pulse allows a simple exponential decay to be added to the steady‐state solution. This is contrary to the approach described by Deoni in 2009 (9), in which the steady‐state magnetization immediately before each RF pulse was considered. The $e^{-T_{E}/T_{2}^{\prime }}$ term is present in the SPGR and not in the bSSFP sequence model, which is a consequence of the latter's spin‐echo behavior as described in 26.

Equation [1] is only valid if perfect spoiling of the transverse magnetization is attained before each RF pulse. This is easily obtained for $T_{R}\gg T_{1}$ however, RF and gradient spoiling methods need to be applied when $T_{R}\ll T_{1}$. A good discussion of this issue is presented in 27, and the reported value of 50° as “stable” RF phase increment is assumed in this paper; no further correction to the apparent $T_{1}$ obtained is applied. However, the obtained steady state is still $T_{R},T_{1},\;T_{2}$ and FA dependent. To ensure that imperfect spoiling was kept under control, the SPGR sequence was modeled using the extended phase graph (EPG) algorithm 28, 29, 30, including attenuating effects associated with diffusion resulting from both imaging and spoiler gradients 28, 31. Choice of FA and TR was then constrained to ensure a peak error of less than 5% compared with the ideal Ernst regime for relevant T_1_ and T_2_ ranges.

### Cramér‐Rao Lower Bound

Following the notation presented by Lankford and Does 13, considering a model g(**x,θ**), where $x={\left[\alpha_{1},\alpha_{2},\ldots ,\;\alpha_{N}\right]}^{T}$ is a vector containing N independent measurements (in the case of this paper, SPGR and bSSFP images at specific FA and T_R_) and $\theta ={\left[\theta_{1},\theta_{2},\ldots ,\;\theta_{M}\right]}^{T}$ is a vector containing M model parameters (eg, $M_{0}$, $T_{1}$, $T_{2},\;\text{and}\;\Delta \Omega \;(\text{with}\;M=4)$ then the variances of the elements of the unbiased estimate vector of the model parameters $\hat{\theta }$ based on noisy measurements $y={\left[y_{1},y_{2},\ldots ,\;y_{N}\right]}^{T}$ are described by the M × M covariance matrix $\Sigma_{\hat{\theta }}^{2}$ as follows:

(6) $$\Sigma_{\hat{\theta }}^{2}\geq \;F^{-1}$$

where *F* is the Fisher information matrix. The CRLB, which is the lowest limit of $\Sigma_{\hat{\theta }}^{2}$ is obtained by taking the equality in Equation [6].

Assuming y follows a Gaussian noise distribution with standard deviation σ, *F* simplifies 13, 32 to:

(7) $$F_{j,k}=\overset{N}{\underset{i=1}{\sum }}\frac{1}{\sigma_{i}^{2}}\frac{\partial g_{i}}{\partial \theta_{j}}\frac{\partial g_{i}}{\partial \theta_{k}}$$

where j and k (*j, k* = 1, 2,…, *M*) are, respectively, the row and column indexes of *F*. The CRLB has been used previously to optimize experiment design in diffusion MRI 33 and quantitative magnetization transfer MRI 16. Following the methodology proposed in 16, we sought to enhance the estimation precision for multiple parameter estimates (eg, $T_{1}$ and $T_{2}$ by considering a subset, L of diagonal elements of $\Sigma_{\hat{\theta }}^{2}$ simultaneously, which corresponds to only considering the minimal obtainable variance of each parameter of interest.

To achieve equal relative precision (defined throughout this work as $p_{\theta_{m}}^{\text{CRLB}}=\text{diag}{\left(\Sigma_{\hat{\theta }}^{2}\right)}_{m}/\theta_{m}^{2}$ for each degree of freedom, the CRLB of each parameter is weighted by the inverse square of the parameter value. This means we can compute a root mean squared precision $P_{rms}^{\text{CRLB}}$ as follows:

(8) $$P_{rms}^{\text{CRLB}}=\overset{L}{\underset{m=1}{\sum }}\sqrt[{}]{\frac{\text{diag}{\left(\Sigma_{\hat{\theta }}^{2}\right)}_{m}\;\;\;}{\theta_{m}^{2}}}=\overset{L}{\underset{m=1}{\sum }}\sqrt[{}]{p_{\theta_{m}}^{CRLB}}$$

It is important to notice that $P_{rms}^{\text{CRLB}}$ is computed for a specific parameter vector **θ** (eg, $M_{0}$, $T_{1}$, $T_{2},\;\text{and}\;\Delta \Omega$) However, we are interested in being able to evaluate $P_{rms}^{\text{CRLB}}$ over a wide range of different **θ**. Therefore, we compute $P_{rms}^{\text{CRLB}}$ over G different parameter vectors $\;\theta_{G}=\left[\theta_{1G},\theta_{2G},\ldots ,\theta_{MG}\right]$ and the maximum value obtained is minimized, resulting in the following cost function, CF_grid_, which is to be minimized as follows:

(9) $$CF_{grid}=\underset{G}{\max}\left\{P_{rms}^{\text{CRLB}}\left(\theta_{G}\right)\right\}$$

CF_grid_ is conservative in that it always selects the worst‐case relative precision detected on the defined search space grid, and therefore minimizes the estimation variance for the worst case in all of the $\;\theta_{G}$ considered.

## METHODS

All simulations and offline postprocessing were performed using MATLAB 2016a (The MathWorks Inc, Natick, MA, USA). Equations [1] to [5] were normalized by $M_{0}$ effectively defining them with respect to unit proton density. To fit the JSR model, which is the concatenation of the SPGR and bSSFP signal models established in Equations [1] to [5], to actual image data, it is necessary to take account of the strength of the signals found in each voxel. This is proportional to $M_{0}$ scaled by an arbitrary position‐dependent complex gain $\;Ae^{-i\phi_{A}}$ which can be attributed to receive sensitivity, including all sources of incidental phase.

(10) $$\text{JSR}=Ae^{-i\phi_{A}}M_{0}\left[S_{\text{SPGR}}^{1},\ldots ,S_{\text{SPGR}}^{N_{SPGR}},S_{\text{SSFP}}^{1},\ldots ,S_{\text{SSFP}}^{N_{\text{bSSFP}}}\right]$$

Because it is not possible to separate the different contributions to this compound scaling factor, we define a complex “weighted proton density” $\rho =Ae^{-i\phi_{A}}M_{0}=\rho^{r}+i\rho^{i}$ This formulation keeps the bSSFP model and acquisitions everywhere differentiable, which is not the case in their characteristic stop‐band areas when using magnitude images, allows ΔΩ to be directly estimated from the image data, and retains a Gaussian image noise distribution. The SPGR acquisitions may contain additional phase factors, which might require an extra parameter to fit that is not needed for relaxation‐time determination. We therefore discard the SPGR image phase by taking the magnitude and enforcing $\rho_{SPGR}=\left|\rho \right|=\sqrt[{}]{{\left(\rho^{r}\right)}^{2}+{\left(\rho^{i}\right)}^{2}}$ resulting in the final considered JSR model of Equation [11]:

(11) $${\text{JSR}}_{\text{final}}=\left[\left|\rho |S_{\text{SPGR}}^{1},\ldots ,\left|\rho \right|S_{\text{SPGR}}^{N_{SPGR}},\;\;\text{Re}\left(\rho S_{\text{SSFP}}^{1}\right),\text{Im}\left(\rho S_{\text{SSFP}}^{1}\right),\;\ldots ,\text{Re}\left(\rho S_{\text{SSFP}}^{N_{\text{bSSFP}}}\right),\text{Im}(\rho S_{\text{SSFP}}^{N_{\text{bSSFP}}}\right)\right]$$

where real (Re) and imaginary (Im) signals are concatenated to keep the cost function real valued. For this approach, it is important that the signal‐to‐noise ratio (SNR) of each SPGR image set be sufficient to avoid significant Rician bias. This was confirmed empirically by using the MATLAB Kolmogorov‐Smirnov test to check for normal distributions on white matter (WM) and gray matter (GM) segmented SPGR images.

For both simulation and optimization, a grid of T_1_, T_2_ pairs were used to cover the relevant ranges for brain (obtained from pilot data acquired while setting up this study), from 600 to 1200 ms in steps of 25 ms for T_1_ and 25 to 80 ms in increments of 5 ms for T_2_. Although estimated, cerebral spinal fluid relaxation times have been excluded from the optimization, as it is difficult to guarantee correct spoiling of the magnetization and are, usually, of less clinical interest. Optimizing the measurement to estimate the higher T_1_, T_2_ values of cerebrospinal fluid (CSF) could diminish precision for brain tissue, while not achieving any valid measurement. The proton density was given the value of 10 in arbitrary units with zero phase (ie, $\rho^{r}=10\;\text{and}\;\rho^{i}=\;0i$). Although not necessary, for ease of guaranteeing equal sampling conditions between acquisitions such as bandwidth and geometrical distortions, $T_{E}^{SPGR}=T_{E}^{SSFP}=0.5T_{R}^{SSFP}$ was imposed. Also, because of the short readout times used in this work, SPGR signal $T_{2}^{\prime }$ deviations were neglected. This is justified, because, for typical frontal WM of $T_{2}^{\prime }=285\;\text{ms}$ 34, induced signal deviations of not considering $e^{-T_{E}^{SPGR}/T_{2}^{\prime }}$ are less than 1%.

All images were obtained on a 3 Tesla (T) Philips Achieva‐TX system (Philips Healthcare, Best, Netherlands) with the manufacturer 32‐channel receive adult head coil and processed from k‐space raw data using the *MRecon* environment (Gyrotools LLC, Zurich, Switzerland). To assure equal RF pulse duration T_RF_ for all acquired sequences, which allows a common finite pulse width correction 8, 25 to be used, the scanner software was modified to force a fixed pulse duration that is determined by the largest FA required with all other FA obtained by varying the pulse amplitude only. Nonlinearity of the RF amplifier was corrected by enforcing a vendor calibration step, which allows pre‐emphasis of the RF shape. The VFA data were sampled as a sagittal acquisition with a field of view (FOV) of 250 × 250 × 250 mm^3^ at 0.8‐mm^3^ isotropic resolution, fixed bandwidth of 959 Hz/pixel, and SENSE factor of 2 (anterior–posterior) and 2 (right–left) in both phase‐encode directions for both in vivo and phantom measurements. The SPGR spoiling area was kept at the default values optimized by the manufacturer software, which resulted in a total phase dispersion of approximately 7 radians per voxel in the readout direction. Nonselective excitation pulses were used for all 3D measurements. Correct knowledge of transmit field (B_1_) distribution is assumed as it can be assessed with mapping techniques 31, 35, 36, 37, which are not the main focus of this paper. The spatially dependent transmit scaling factor, k (defined in Eqs. [(1), (2), (3), (4), (5)]), was experimentally measured using the actual flip angle approach 37, with a TR_1_/TR_2_ = 25/125 ms and maximum allowed gradient spoiling between each TR. The FOV was set to 250 × 250 × 250 mm^3^ for an acquired isotropic resolution of 3.91 mm^3^, resulting in a total acquisition time of 2 min. The actual FA, $\alpha_{actual}$ was then specified as $k\alpha_{prescribed}$ in all calculations.

The parameter estimates, $\theta_{JSR}=\left[\rho^{r},\rho^{i},{\;T}_{1},T_{2}\text{ and}\;\Delta \Omega \right]$ for each measured voxel are obtained through fitting Equation [11] on a least‐squares criteria using the MATLAB 2016a *lsqnonlin* routine. The objective function was defined as the sum of the square difference between the model and the measured signal intensity, and the stopping criteria were set as $1\times 10^{-15}$ tolerance on the cost function value or a maximum of 500 iterations. The optimization's initial conditions were kept fixed for all estimated voxels and chosen as the expected average relaxation time in the entire brain from pilot data acquired while setting up this study. For all experimental estimations, each imaged voxel was independently processed on an Intel Xeon CPU E5‐2687W 0 at 3.10 GHz, parallelized to 16 cores, taking a total computational time of between 6 and 7 h, depending on the extracted brain volume.

For comparison purposes, linearized estimations of $\rho_{1}^{\prime },{\;T}_{1}$ were obtained using the DESPOT1 approach as described in 6, and then $\rho_{2}^{\prime },{\;T}_{2},\;\text{and}\;\Delta \Omega$ were estimated using DESPOT2‐FM 9 with a finite RF pulse correction 8, 25. Note that in this approach the proton density is separately estimated for each type of image, requiring the introduction of $\rho_{1}^{\prime }\;$ and $\rho_{2}^{\prime }$ As a point of reference for the optimized acquisition, a baseline DESPOT1/2 protocol was adapted from 9 and consists of two SPGR (DESPOT1 ‐ > FA = 4 ° and 18 °, TR = 6.2 ms) and four bSSFP (DESPOT2 ‐ > FA = 15 ° and 65 °, TR = 4.2 ms for $\phi_{\text{RF}}=\pi ,0\;\text{rad}$ measurements resulting in a total acquisition time of 9 min and 18 s.

### CRLB Numerical Validation

Validation that the CRLB is able to accurately predict JSR estimation quality was performed with a Monte Carlo simulation using the previously defined values of ρ, T_1_, and T_2_. For each element of the resulting grid, $1\;\times \;10^{5}$ independent trials were generated. Gaussian distributed noise with zero mean and a standard deviation σ=0.02|ρ|  13was added to both real and imaginary parts of each signal.

For each $T_{1}$ and $T_{2}$ combination, the standard deviation σ of the Monte Carlo simulation as well as the predicted CRLB, $\sigma_{\text{CRLB}}$ for both relaxation times were extracted. For comparison, each value was normalized by its respective relaxation‐time defining Monte Carlo and $\sigma_{CRLB}^{2}$ precisions as $p_{T_{1,2}}^{MC}=\surd (\sigma_{T_{1,2}}^{2}/T_{1,2}^{2})\;$d $p_{T_{1,2}}^{CRLB}=\surd (\sigma_{CLRB}^{2}{}_{T_{1,2}}/T_{1,2}^{2})$, respectively.

### Phantom Validation

Phantom validation was performed by imaging an in‐house built spherical phantom filled with a 0.5% agarose, 0.9% NaCl, and 0.02‐mM MnCl_2_ solution, and primarily focused on two goals:

1. Validation of estimation improvement based on JSR fitting approach; and
2. Validation of the CRLB framework as a protocol design tool.

Comparison between the conventional two‐step fitting approach and the proposed JSR is performed using the baseline protocol. Both the DESPOT1/2 and JSR estimation maps were obtained using the same measured data with the procedure described previously. Reference relaxation values for the phantom were obtained using spin‐echo measurements within a single slice with a FOV of 250 × 250 mm^2^ and an acquired voxel size of 1.6 × 1.6 × 4 mm^3^. The $T_{1}$ was mapped using single‐shot inversion recovery fast spin echo with k‐space filled from low to high frequencies (minimizing T_2_ effects) for inversion times of 107 to 1857 ms in increments of 125 ms. Measurements were separated by a 20‐s gap to guarantee full $T_{1}$ recovery. The $T_{2}$ was obtained by multi‐echo spin echo sampled at 32 echoes (each echo samples an image with different T_2_‐weighted contrast) with echo times ranging from 15 to 480 ms in increments of 15 ms and a fixed TR of 2000 ms. To minimize imperfect refocusing contributions, only the even echoes were used to estimate $T_{2}$ decay, and both $T_{1}$ and $T_{2}$ maps were estimated based on a least‐square criteria against their expected mono‐exponential decay curves 3, 22. Although spin‐echo methods may sometimes be described as “gold standard,” in the present context we note that they provide a robust means of estimating relaxation parameters by a completely different approach to the one under investigation, and therefore provide an independent point of reference. However, this distinctiveness may also lead to discrepancy with the family of methods under investigation. The magnetization transfer ratio (MTR) was also measured using the standard Philips product sequence ‐ transverse acquisition with voxel size of 1 × 1 × 2 for a FOV of 224 × 168 × 120, sinc‐shaped preparation with 19.3‐ms duration, max amplitude of 12.2 µT, and off‐resonance of 1100 Hz.

To validate the proposed CRLB framework, a phantom‐specific VFA protocol was designed and the resulting parameter estimation was compared with the baseline protocol defined previously.

### Optimizing JSR

This work proposed that the CRLB can be used as a criterion to select the optimal JSR acquisition parameters FA, T_R_, and $\phi_{\text{RF}}$. To achieve this, Equation [9] was minimized making use of the pattern search optimization routine, implemented in the MATLAB 2016a *optimtool*. The standard $T_{1}$ and $T_{2}$ grid defined previously was combined with field offsets ranging from −125 to 125 in 1 Hz increments. This routine is not guaranteed to find global optima, so the optimization was simply repeated for 10 randomly distributed starting values for FA, T_R_, and $\phi_{\text{RF}}$ The global optimum, if not obtained, is not expected to dramatically improve the estimation quality compared with the solution achieved, as in practice, several different local minima are found whose cost function demonstrate similar performance.

A key constraint that must be considered is the total imaging time, as it is always possible to improve performance simply by imaging for longer (ie, at the expense of efficiency). Working at fixed resolution (taken to be the same for all of the contributing acquisitions) with only one average, the total imaging time is proportional to the sum of the repetition times of the individual sequences in the protocol. For the considered baseline protocol 9, $T_{\text{total}}=29.2\text{ ms}$ was taken as a fixed upper time limit for all solutions explored. Within this constraint, the number of SPGR (N_SPGR_) and bSSFP (N_bSSFP_) measurements can be varied along with the TR, $\phi_{\text{RF}}$ and FA for each acquisition. Solutions can be calculated using different values of TR for each acquisition, however, early exploratory work showed that the optimal acquisitions converged for $T_{R}^{\text{bSSFP}}=\min\left\{T_{R}\right\}$ thus, for simplicity, we constrained bSSFP to have minimum $T_{R}$. This time depends on the resolution, and particularly FA (specific absorption rate constraints), but is otherwise the same for both SPGR and bSSFP on our system. Allowing for a suitable range of FAs, we set $T_{R_{\text{min}}}=4.2\;\text{ms}$ which sets the maximum possible number of acquisitions in the permitted T_total_. The $T_{R}^{\text{SPGR}}$ is allowed to freely vary, subject to $\sum^{}T_{R}^{\text{SPGR}}+N_{\text{bSSFP}}T_{R}^{\text{bSSFP}}\leq T_{\text{total}}$ in which an explicit sum is written because both the number of SPGR sequences and their individual repetition times are varied.

With five unknowns to be estimated $\theta_{JSR}=\left[\rho^{r},\rho^{i},{\;T}_{1},T_{2}\text{ and}\;\Delta \Omega \right]$ a minimum of five measurements are required for a fully determined solution. In addition, given that equal echo times are used for all acquisitions, a minimum of two bSSFP measurements is required to estimate the field map ΔΩ.

The optimal $T_{R}^{\text{SPGR}}$, $\phi_{\text{RF}}$ and FAs were therefore determined for different combinations of N_bSSFP_ and N_SPGR_, as expressed in Table 1 (grayed‐out cells are excluded as infeasible).

**Table 1:** Optimization Options Explored within the Time Constraint Explored

		N_bSSFP_				
		1	2	3	4	5
N_SPGR_	**1**				✓	✓
	**2**			✓	✓	✓
	**3**		✓	✓	✓	
	**4**		✓	✓		

Note: Gray areas were not considered in the optimization search, as they are infeasible.

### In Vivo Validation

In vivo scans were acquired in four healthy volunteers (two male, two female, mean age 27 (min = 20, max = 31)), who gave written informed consent according to local ethics requirements.

Each subject was imaged using the complete baseline protocol and an optimized set of parameters were obtained by making use of the proposed CRLB optimization framework. The optimized parameters are listed in the “Results” section.

Before relaxation‐map estimation, all images were skull‐stripped and aligned using standard FSL bet and flirt tools (www.fsl.fmrib.ox.ac.uk) 38, 39, 40. An automatic segmentation was also performed on the T_1_‐weighted SPGR image using the FSL *fast* tool (38‐40).

Individual T_1_ and T_2_ histograms were calculated to compare the estimation performance between baseline and optimized protocols 3, 41.

## RESULTS

### CRLB Numerical Validation

**Figure 1:**
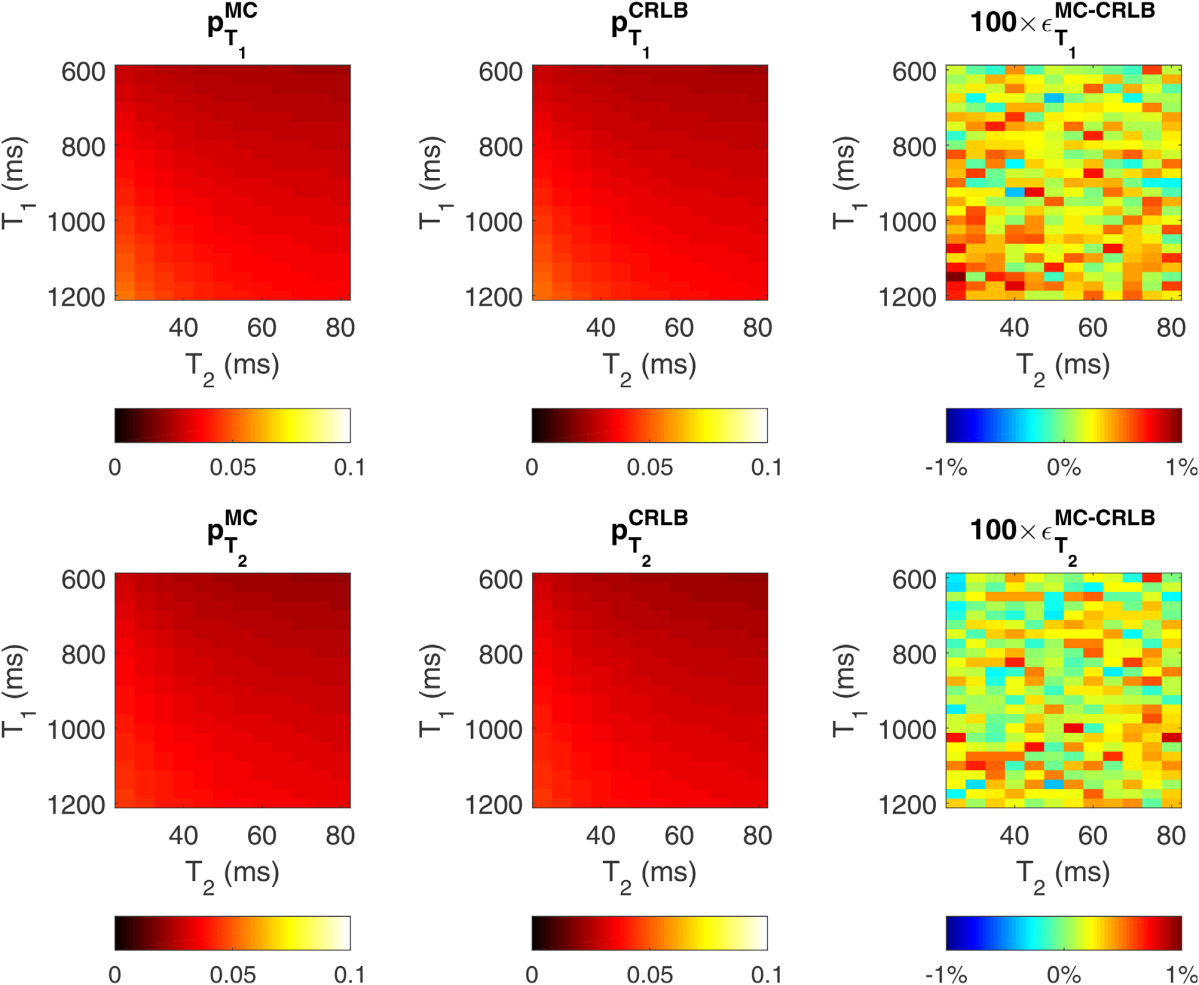
Comparison between $\sigma_{CRLB}^{2}$ (left column) and Monte Carlo simulations (middle column) predicted precisions for different T_1_ and T_2_ relaxation‐time combinations. The right‐hand column displays the percent different between $p_{T_{1}}^{\text{CRLB}}$ and $p_{T_{1}}^{\text{MC}}$ predictions ( $\epsilon_{T_{1}}^{\text{MC}-\text{CRLB}}=(p_{T_{1,2}}^{\text{MC}}-p_{T_{1,2}}^{\text{CRLB}})/p_{T_{1,2}}^{\text{CRLB}}$)

Figure 1 shows a comparison of the JSR CRLB $p_{T1,2}^{CRLB}$ and Monte Carlo–predicted precision $p_{T1,2}^{\text{MC}}$ for the baseline protocol of the full grid of brain relaxation times. The relative percentage difference between $p_{T_{1,2}}^{CRLB}$ and $p_{T_{1,2}}^{\text{MC}}$ and defined as $\epsilon_{T_{1}}^{\text{MC}-\text{CRLB}}=(p_{T_{1,2}}^{\text{MC}}-p_{T_{1,2}}^{\text{CRLB}})/p_{T_{1,2}}^{\text{CRLB}}$ is below 1%, as can be observed in the right column of Figure 1.

### Phantom Validation

**Figure 2:**
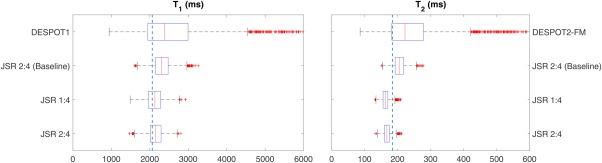
Box and whisker plots comparing T_1_ and T_2_ distributions within a 10‐mL region of interest, estimated using each of the acquisition protocols defined in Table 2. Each acquisition protocol is identified by the number of SPGR:bSSFP measurements. Baseline corresponds to the acquisition protocol adapted from (9). Vertical lines show the average spin echo–measured T_1_ and T_2_. Parameter estimation by DESPOT was processing for the baseline protocol only or JSR, as indicated.

The phantom had T_1_ = 2058 ± 27 ms and T_2_ = 185 ± 1 ms, as determined using the reference spin‐echo sequences and MTR = 5.8% ± 0.7%. Phantom data were used to validate the hypothesis that JSR precision outperforms the standard DESPOT1/2 approach, and allowed the proposed CRLB framework to be tested. To achieve this, an extensive set of phantom‐specific optimized acquisition protocols (Table 1) were obtained using the CRLB framework, and the two best protocols are reported in Table 2, together with the baseline parameters for comparison. These three protocols were acquired in the same scanning session, and the respective T_1_ and T_2_ distributions inside a 10‐mL region at the center of the phantom are compared in the box plots of Figure 2. For reference, a blue dotted line is drawn that marks the T_1_ and T_2_ values obtained using spin echo–based estimation.

**Table 2:** Phantom‐Specific Acquisition Protocols $T_{R}^{bSSFP}=4.2ms$

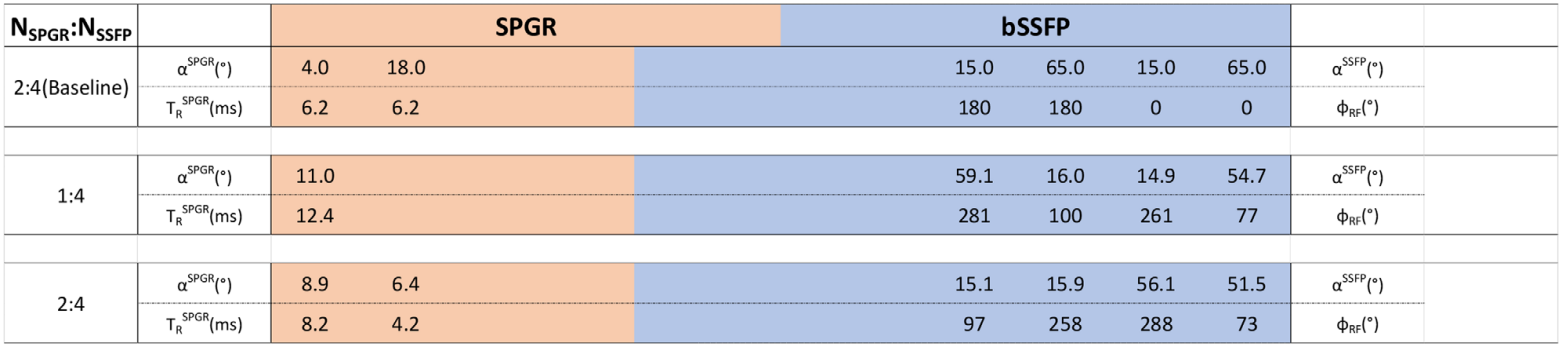

Note: Color bars represent the fraction of time that SPGR (orange) and bSSFP (blue) occupy in each protocol. $T_{R}^{bSSFP}=4.2\;\text{ms}$ in all cases. The JSR‐optimized solutions were obtained for the expected T_1_ and T_2_ values and independently assessed using spin‐echo methods. For each protocol, the FAs are listed in the upper row, and the bottom row lists the repeat times, for the SPGR and the phase increments for the bSSFP sequences, as indicated by the adjacent labels. Only JSR 1:4 and 2:4 are displayed, as they were the best two performing solutions from the explored set (Table 1).

The conventional two‐step fitting approach on the baseline protocol results in interquartile range estimations of 1162 ms for DESPOT1 T_1_ and 111 ms for DESPOT2‐FM T_2_. Significant improvement is achieved by performing the proposed JSR approach on the same data, with interquartile ranges reduced to 323 ms for T_1_ and 26 ms for T_2_. Further improvement is obtained by making use of the proposed CRLB optimization framework. The JSR‐1:4 and JSR‐2:4 interquartile T_1_ ranges are reported as 220 and 200 ms (32 and 38% reductions), respectively. Similar improvement can be observed in T_2_ estimation, in which the JSR‐1:4 and JSR‐2:4 interquartile ranges are 14 and 15 ms (46 and 43% reductions), respectively.

The percentage deviation of the median value relative to the reference $T_{1}$ spin‐echo measurement DESPOT1, JSR baseline, JSR 1:4, and JSR 2:4 demonstrate 15.1, 11.0, 3.3, and 4.4% bias, respectively. On the same note, the $T_{2}$ percentage deviation is reported as 20.7% for DESPOT2‐FM, 10.9% for JSR baseline, −11.4% for JSR 1:4, and −9.2% for JSR 2:4.

### Optimizing JSR

**Figure 3:**
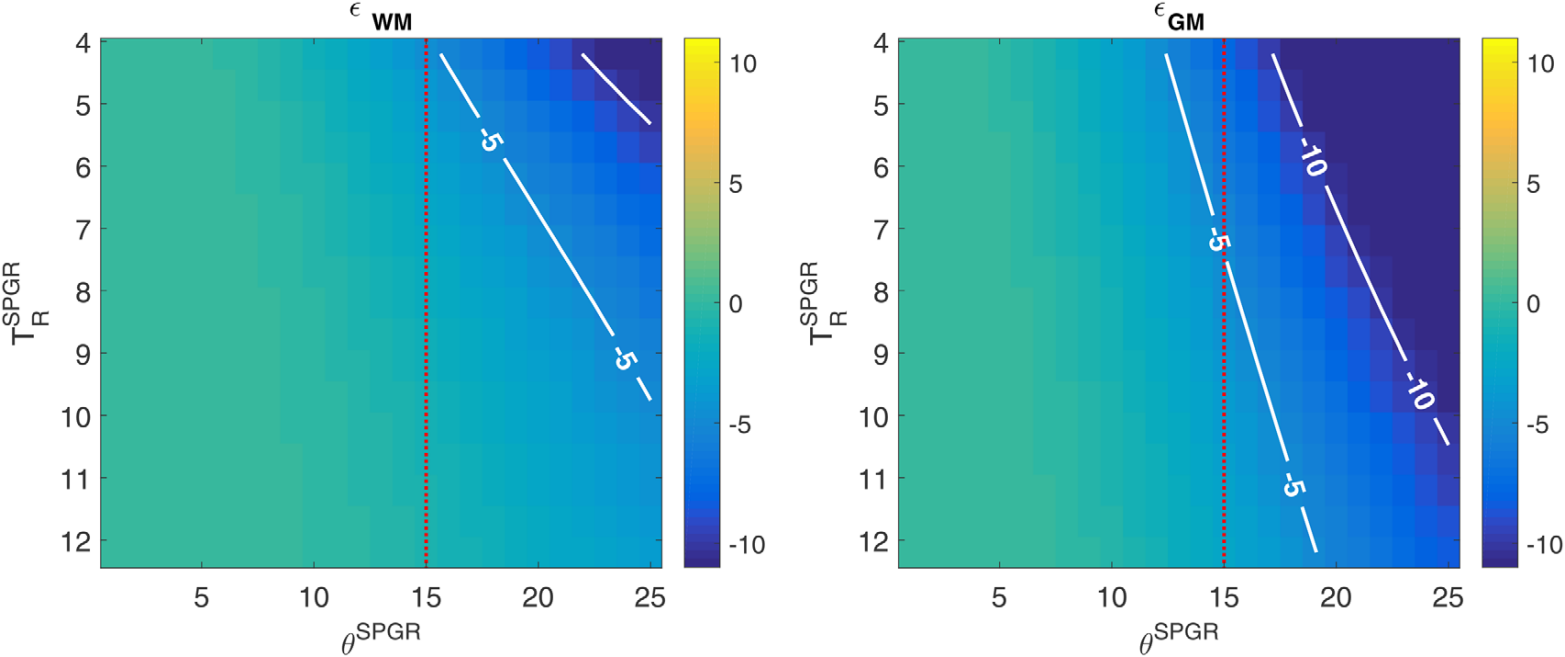
Percentage deviation error $\epsilon =(S_{spgr}-S_{spgr}^{EPG})/S_{spgr}^{EPG}$ between EPG and Ernst model for both WM ( $\epsilon_{\text{WM}}\;$) and ( $\epsilon_{\text{GM}}$) as functions of repeat time and FA for the SPGR sequence. The red dotted lines represent the maximum allowed SPGR FA that is consistent with ensuring the error for WM is kept below 5%.

The SPGR acquisitions have been criticized for their sensitivity to imperfect spoiling of transverse magnetization before each excitation pulse 3, 36, 42. To mitigate this, EPG simulation of the SPGR signal response for the parameters of interest was compared with the signal intensity predicted by the Ernst equation. Figure 3 demonstrates the expected percentage signal error $\epsilon =(S_{spgr}-S_{spgr}^{EPG})/S_{spgr}^{EPG}$ for both WM ( $\epsilon_{\text{WM}}$) and GM ( $\epsilon_{\text{GM}}$) as a function of different $T_{R}^{\text{SPGR}}$ and FA $\alpha_{\text{SPGR}}$ To avoid solutions with a WM error larger than ±5%, the maximum allowed FA was set to 15^o^ (red line in Fig. 3).

As defined in the “Methods” section, optimized solutions were sought using the CRLB framework for the different combinations of SPGR and bSSFP measurements presented in Table 1. The results can be seen in Table 3, which is color‐coded based on the fraction of time spent on either SPGR (orange) or bSSFP (blue) acquisitions. Each bar shows the parameters obtained for each explored combination of N_SPGR_:N_bSSFP_ measurements, and is rank‐ordered based on their expected CF_grid_ value (Eq. [9]; last column in Table 3). All explored protocols are restricted to a self‐imposed total time constraint of T_total_ = 29.2 ms.

**Table 3:** Brain‐Specific Optimized JSR Acquisition Protocols $T_{R}^{bSSFP}=4.2ms$

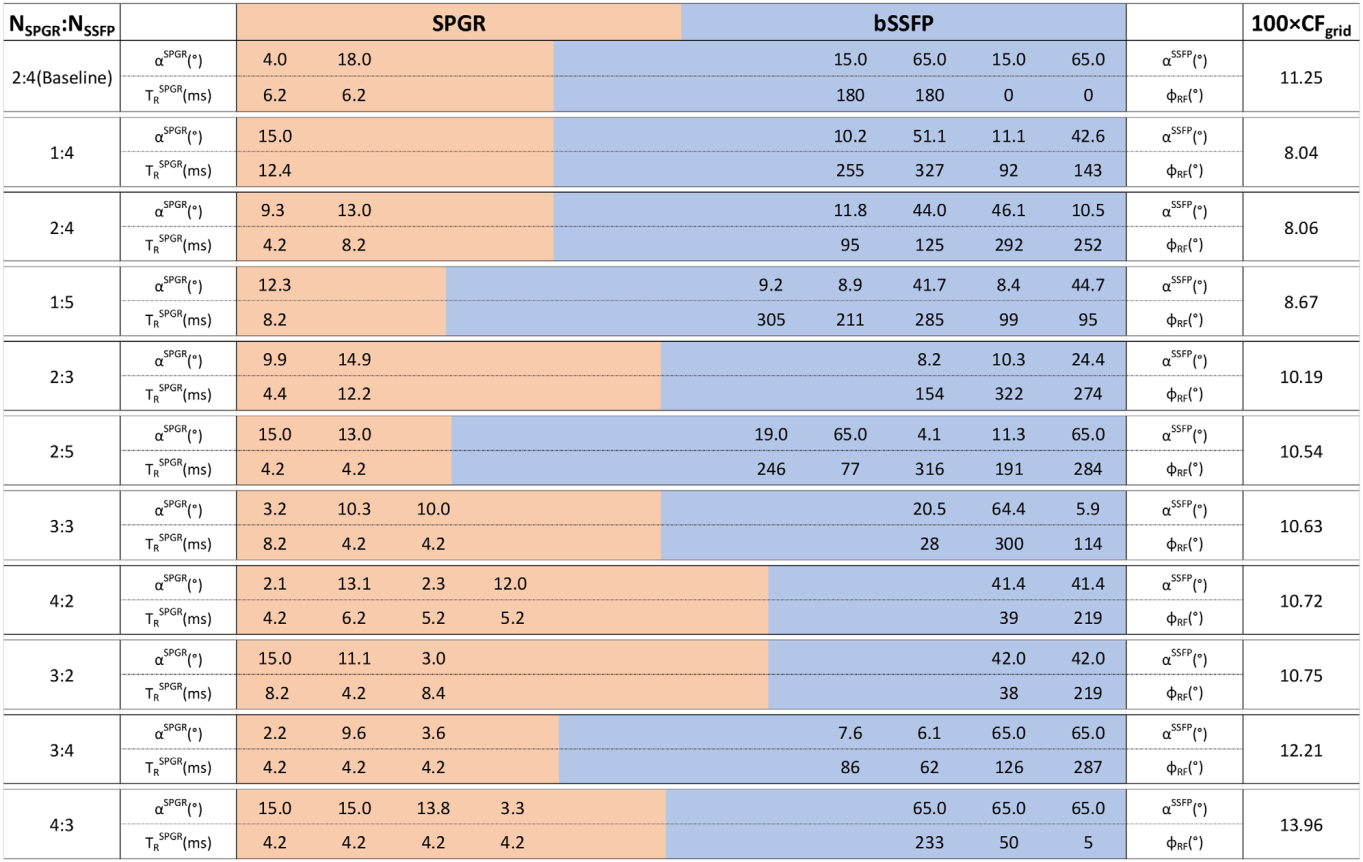

Note: Baseline shown for reference. The 2:4 acquisition protocol was selected for in vivo acquisition. From top to bottom, optimized protocols are rank‐ordered based on their attained worse‐case percentage precision (100 × CF_grid_ − eq. 9) values. Each row is color‐coded based on the fraction of time spent on either SPGR (orange) or bSSFP (blue) acquisitions. All obtained protocols are subject to a T_total_ = 29.2 ms constraint.

### In Vivo Experiments

**Figure 4:**
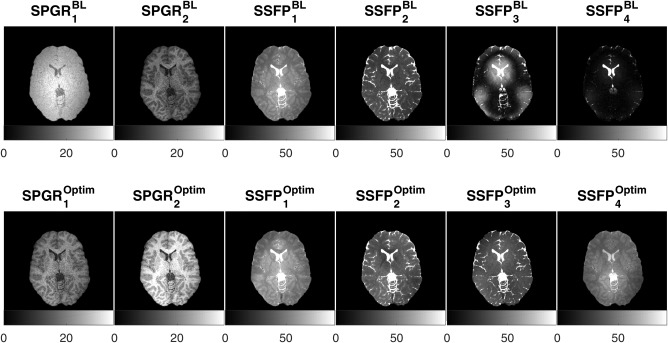
A representative axial slice of in vivo acquired data from the baseline (top row) and optimal (bottom row) protocols. Acquisition parameters for each image are given in Table 3, listed in the same order as presented here.

In vivo validation compared the estimation maps obtained using conventional DESPOT1/2 and JSR fitting methods, with data acquired using the baseline protocol. Based on Table 3, a brain‐optimized JSR protocol with two SPGR and four bSSFP measurements was also acquired. Given the similar performance between 1:4 and 2:4, we chose the 2:4 acquisition, as collecting two SPGR scans provides a more robust solution for subjects who might not be able to be so still. For reference, representative axial slices of the different acquisition parameters are demonstrated in Figure 4.

**Figure 5:**
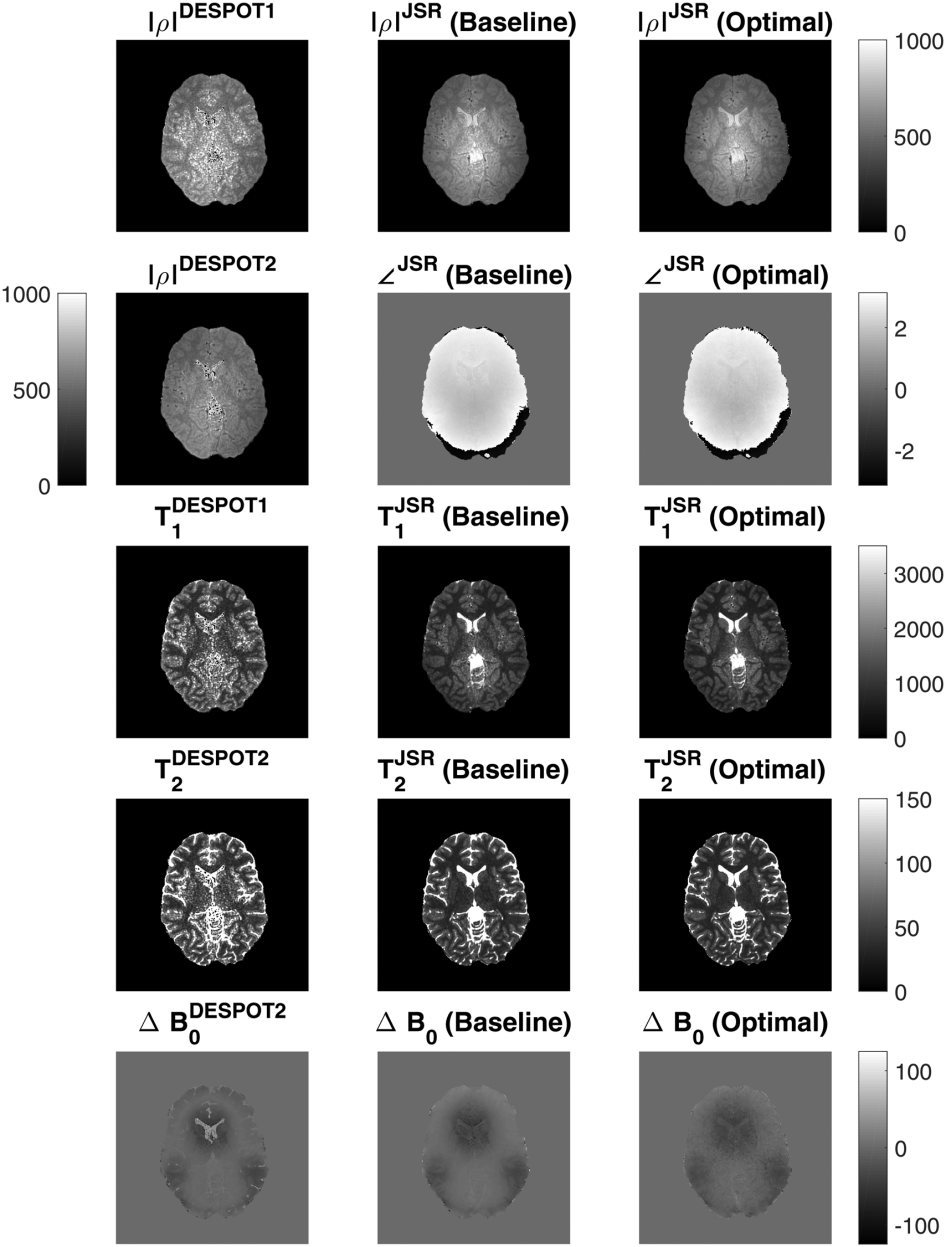
Comparison among conventional DESPOT1/2 approach (left column), JSR with baseline protocol (middle column), and JSR optimized protocol (right column) estimation maps.

Exemplar estimation maps can be seen in Figure 5. Note that DESPOT 1/2 relaxation parameter maps (left column in Fig. 5) are clearly more noisy that the corresponding JSR maps (columns 2 and 3 in Fig. 5). In addition, the off‐resonance map obtained with the two‐step fit is much more contaminated by explicit anatomy (particularly the CSF spaces) than the corresponding estimate from the JSR process.

**Figure 6:**
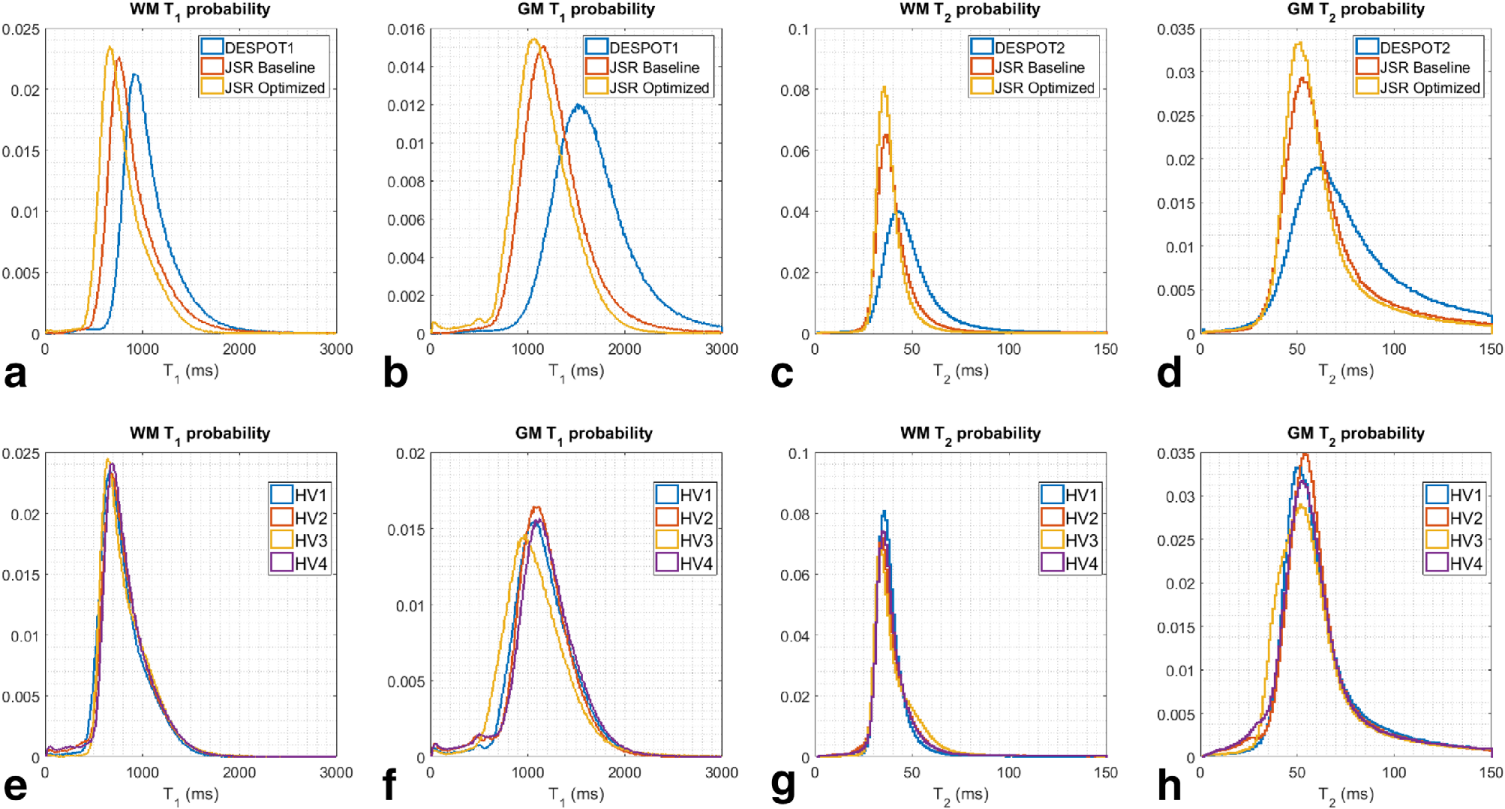
Tissue‐specific histograms for in vivo acquisition. Top row compares the distributions obtained for Healthy Volunteer 2 (HV2) with conventional DESPOT1/2 (blue), JSR with baseline parameters (red), and JSR with optimized acquisition parameters (yellow). Bottom row compares JSR‐optimized histograms for all imaged volunteers. Tissue segmentation was obtained using the FSL *fast* tool.

Because of its high SNR and contrast, the adult optimized SPGR with 8.2‐ms TR was used after brain extraction to segment CSF, WM and GM, as described in the “Methods” section. Tissue‐specific relaxation histograms were then plotted for all of the acquisition schemes and are summarized in Figure 6.

Figures 6a to 6d show the WM/GM T_1_ and WM/GM T_2_ pixel‐parameter‐value‐frequency distributions obtained on Healthy Volunteer 2 (HV2) using DESPOT1/2 (blue), JSR with baseline acquisition (red), and JSR with optimized acquisition (yellow) parameters. As with the phantom data, JSR produces narrower estimation distributions compared with the conventional DESPOT1/2 approach. However, the estimation mean relaxation‐time values show significant shifts toward lower relaxation time values.

Figures 6e to 6h show the WM/GM T_1_ and WM/GM T_2_ histograms obtained using the optimized JSR acquisition for all of the healthy volunteers, demonstrating the reproducibility of the proposed method.

## DISCUSSION

This work aimed to improve the precision of DESPOT1/2 based on the JSR approach, in which all of the key parameters are estimated in a single calculation. Figure 2 shows 72%/77% decrease in interquartile range of T_1_/T_2_ estimation between conventional DESPOT and JSR estimation. This is further corroborated by the in vivo data, in which visual assessment (Fig. 5) shows that the JSR approach reduces the noise of the estimated maps, more specifically in deep GM areas where the SNR is intrinsically lower. Tissue‐specific distributions in Figures 6a to 6d show narrower distributions for JSR compared with VFA DESPOT1/2. This is primarily due to conventional DESPOT estimation being limited by the inherently lower SNR of the SPGR acquisition. The noise distribution of T_1_ then limits T_2_ assessment and does not make full use of the high bSSFP SNR. Our approach makes full use of bSSFP information, diminishing the estimation variance for both relaxation times. Although more narrow, there are systemic differences between the estimated relaxation distributions, which are dependent on the specific set of acquisition parameters used, but these are highly reproducible (Figs. 6e–6h). Numerical simulations using the described single‐pool model suggest that minimal to no difference on the average estimated values should occur between the estimation procedures, even when taking into account errors induced by neglecting $T_{2}^{\prime }$. Indeed, not taking this extra decay into account induces higher JSR‐estimated T_1_ values (opposite of what is seen in Figs. 3 and 6), as the reduction in signal is accommodated as higher saturation of the SPGR curve. Another possible source of error is persistent, residual nonlinearities that may occur in the RF power amplifier. One possible solution is to follow the work presented by Lutti et al 43 and fix the RF amplifier power and vary the RF pulse duration to achieve different FAs. In our work, we opted to keep a fixed pulse duration and performed a vendor calibration step on each subject, to allow pre‐emphasis of the nonlinearities. This approach has the advantage of excitation off‐resonance deviations being maintained consistent between different measurements. Phantom data and in vivo discrepancies are most likely the result of magnetization transfer effects, which were not taken into account by the model used in this work, but are known to strongly affect the signal of bSSFP 44, 45, which is now used to jointly estimate T_1_ and T_2_. To corroborate this, the same phantom protocol was acquired on a gel phantom with 4% agarose solution (measured MTR of 20%). For this phantom, the bias between spin echo and VFA measurements was between 5 and 43% for T_1_, and −18% and 22% for T_2_. This is a larger variation than for the 0.5% agarose experiment summarized in Figure 2, in which the range of obtained bias is 4.4 to 15.1% for T_1_ and − 11.4 to 20.7% for T_2_. In Figures 6e to 6h, the optimal JSR protocol tissue‐specific histograms for each of the healthy volunteers are overlaid for comparison. Excellent agreement is shown among all subjects for WM. Relaxation‐times histogram for GM show slightly larger deviations among subjects, although a good agreement is still present. This is most likely because of differences in the brain‐extraction step of our processing pipeline, although some of the discrepancy can also be attributed to genuine interindividual variance.

The in vivo T_2_ values show a significant underestimation when compared with other studies 46, 47, 48. However, this was not the case for the phantom experiment; in fact, failing to apply the T_RF_ correction (8) for the phantom data resulted in an overestimation of the obtained T_2_ values by 15 to 20% compared with the ones displayed in Figure 2. This gives confidence that the correction is appropriate, at least for the phantom, which has a much lower MTR than adult brain 4, 49, although it is still not zero.

The second part of this work focused on the validation of the proposed CRLB framework as a protocol design tool. Numerical validations (Fig. 1) suggest good agreement between the proposed estimation procedure variance and the predicted CRLB (percentage difference < 1%), without affecting the estimation bias, giving confidence in both the fitting routine and the optimization tool. To make all results comparable, and especially because varying the total acquisition time changes the overall SNR in all scenarios, we optimized the SPGR and bSSFP measurements subject to a self‐imposed time constraint T_total_ = 29.2 ms, which matches the baseline protocol taken from the literature 9. Sensitivity of the SPGR measurement to imperfect spoiling was avoided by restricting the maximum allowed SPGR FA to 15^o^. This cut‐off guarantees that the expected WM spoiling inconsistencies are below 5%, whereas GM are kept bellow 8% for all optimization protocols explored. Although some solutions might benefit from allowing higher SPGR FA, this would imply either exploring optimal ways to weight Equation [9], given the EPG versus SPGR deviations, or using a better spoiling scheme such as the one presented in 50. Both solutions are outside the scope of this work, as the two best protocols obtained are expected to induce a signal bias of less than 3% for both WM and GM. Table 1 summarizes the different acquisition options explored in this paper; their respective cost function (CF) figures of merit are summarized in Table 3. The standard approach requires a minimum of two SPGR measurements to estimate T_1_; we note that performing JSR allows the acquisition to require only one SPGR measurement. This is interchangeable with increasing the number of SPGR measurements, provided that the same time is spent sampling SPGR information (Tables 2 and 3), which is further validated in Figure 2, where experimental data corroborate this interchangeability. Both optimal protocols result in a T_1_ and T_2_ interquartile‐range reduction of 81 and 87%, respectively, in comparison to the two‐step fitting process, and 32 and 43% relative to the JSR fitting using the baseline protocol. Allowing a single SPGR measurement could be of significant practical importance, as SPGR acquisitions are routinely used for clinical diagnosis. Therefore, full brain relaxometry can be achieved by adding four bSSFP measurements, which corresponds to less than 5‐min extension of the total scan time.

The JSR framework presented can help achieve optimal acquisition efficiency. In this study, full 3D 0.8‐mm^3^ isotropic relaxation maps of the human brain were obtained with a total acquisition time of 11 min and 18 s (9:18 for SPGR and bSSFP data, plus 2:00 for B_1_ field estimation). Although we restricted our acquisition time to a baseline protocol for comparison, it would be possible to trade off the JSR gain in precision for further reduction of the acquisition time.

The work presented here is a proof of concept and demonstrates the benefit of simultaneously using images with different signal responses to minimize variance in relaxometry estimation. Although neglected throughout this work, the JSR framework can be expanded to estimate $T_{2}^{'}$ by allowing different echo times between acquisitions. In addition, it is feasible to include B_1_ field estimation directly from the VFA data by incorporating an inversion recovery sequence (eg, MPRAGE) into the joint system model. Investigation of such protocols showed that the CRLB design framework can be applied, but initial in vivo testing with JSR processing resulted in T_1_ values with higher systematic deviations for different acquisition parameters (eg, inversion time, RAGE block duration), compared with what is presented in this work. We attribute this to the very different RF conditions engendered by such sequences, causing a model bias as a result of MT effects that are not considered (data not shown). This is currently the subject of further investigation; in particular, the option to explicitly include MT in the JSR approach is an interesting future possibility. The JSR approach has a lot in common with the recently introduced fingerprinting concept (MRF) 51, 52, in that all acquired data are used to estimate the final relaxation and associated parameters. The relative merits of this optimized framework and MRF, both in terms of precision and efficiency, remain to be explored.

## CONCLUSIONS

This work shows an immediate benefit of the proposed JSR analysis approach, compared with conventional DESPOT1/2, in producing relaxation maps with improved precision. It also shows that further improvement can be achieved by making use of the CRLB as a protocol design tool. With this approach, it is possible to achieve submillimeter maps of $\rho ,T_{1},T_{2},\;\text{and}\;B_{0}$ in an 11‐min examination, making the approach appealing for potential clinical use. For examinations in which the clinical protocol already includes SPGR, the additional time needed to achieve quantitative relaxometry maps is even smaller ( < 7 additional minutes, including B_1_ calibration in the example shown). As with all variable FA approaches, the absolute values of the relaxation times found tend to deviate systematically from spin echo–based measurements. However, high reproducibility combined with efficiency endow these methods with significant advantages for larger‐scale studies. The proposed framework enhances both precision and efficiency, further adding to their potential utility.
